# High-Purity Bioactive Ingredient—3*S*,3′*S*-Astaxanthin: A New Preparation from Genetically Modified *Kluyveromyces marxianus* without Column Chromatography and Gel Filtration

**DOI:** 10.3390/antiox12040875

**Published:** 2023-04-04

**Authors:** Wei-Cheng Yuan, Tung-Ying Wu, Pei-Yi Chu, Fang-Rong Chang, Yang-Chang Wu

**Affiliations:** 1 Graduate Institute of Natural Products, Kaohsiung Medical University, Kaohsiung 807, Taiwan; b022020026@gmail.com; 2 Department of Biological Science & Technology, Meiho University, Pingtung 912, Taiwan; x00011700@meiho.edu.tw; 3 Department of Food Science and Nutrition, Meiho University, Pingtung 912, Taiwan; 4 Chinese Medicine Research and Development Center, China Medical University Hospital, Taichung 404, Taiwan; ositachucmu@gmail.com; 5 Department of Marine Biotechnology and Resources, National Sun Yat-sen University, Kaohsiung 804, Taiwan; 6 Drug Development and Value Creation Research Center, Kaohsiung Medical University, Kaohsiung 807, Taiwan; 7 Department of Medical Research, Kaohsiung Medical University Hospital, Kaohsiung 807, Taiwan; 8 Graduate Institute of Integrated Medicine, China Medical University, Taichung 404, Taiwan; 9 Department of Medical Laboratory Science and Biotechnology, Asia University, Taichung 413, Taiwan

**Keywords:** enzyme-assisted extraction, magnesium perchlorate, ORAC, salt-assisted liquid-liquid extraction, yeast

## Abstract

A highly efficient methodology for bioactive ingredient 3*S*,3′*S*-astaxanthin (3*S*,3′*S*-AST) preparation from genetically modified yeast (*Kluyveromyces marxianus*) with a combination of enzyme-assisted extraction and salt-assisted liquid-liquid extraction (SALLE) was achieved. The highest yield of 3*S*,3′*S*-AST indicated that FoodPro^®^ CBL for yeast cell walls hydrolysis could significantly enhance extraction and obtain, with the help of SALLE procedure, quantified 3*S*,3′*S*-AST over 99% in purity through cation chelation. In the oxygen radical antioxidant capacity (ORAC) assay, the antioxidant capacity of high-purity 3*S*,3′*S*-AST products were 18.3 times higher than that of the original raw material extract. This new combination preparation may replace previous methods and has the potential to be scaled up in the manufacture of high-purity 3*S*,3′*S*-AST from low-value bioresources of raw materials to high-value products in the food and/or drug industries with lower cost and simple equipment.

## 1. Introduction

Astaxanthin (AST), a natural reddish-colored product, is a carotenoid widely found in various marine organisms, e.g., salmon, phytoplankton, and crustaceans [1]. Its unique structure enables strong antioxidant activities. Evidence suggest that AST has health-promoting cytoprotective effects against cancer, diabetes, and neurodegenerative diseases [2]. Furthermore, AST may be beneficial to individuals with degenerative diseases, dyslipidemia, and cardiovascular diseases [3]. The huge commercial potential in a variety of markets, i.e., cosmetics, nutraceutical, and pharmaceutical, has led a trend of AST products. With a significant amount of research reporting the beneficial bioactivities of AST for human health, there is now a need to design a method for AST preparation that is expected to fit the market demand. Therefore, the development of preparation methods for high-purity and high-value 3S,3′S-AST attracts a lot of attention and interest.

**Figure 1:**
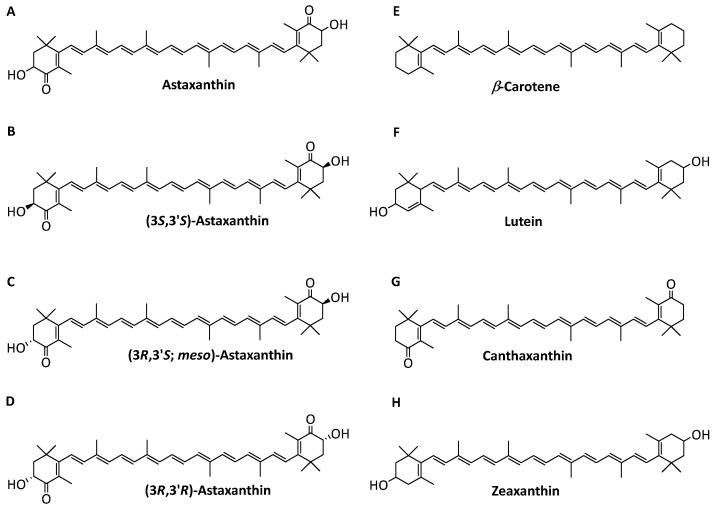
Structure of carotenoids and astaxanthin isomers: (**A**) astaxanthin, (**B**) (3*S*,3′*S*)-astaxanthin, (**C**) (3*R*,3′*S*; meso)-astaxanthin, (**D**) (3*R*,3′*R*)-astaxanthin, (**E**) *β*-carotene, (**F**) lutein, (**G**) canthaxanthin, and (**H**) zeaxanthin.

AST occurs as three optical isomers, (3*S*,3′*S*), (3*R*,3′*R*), and (3*R*,3′*S*) (Figure 1A–D), which show compositional differences and are found in various natural sources [4]. The (3*S*,3′*S*-) isomer is the major component in *Haematococcus pluvialis* [5]. *H. pluvialis* is considered the best resource of natural AST, and it contains 2–4% AST in *Haematococcus* under some severe conditions [6]. Due to a high production, it was the commercial microalgae that was first applied for industrial-scale production of natural AST [7]. Chemically, an efficient organic synthesis pathway from isophorone, cis-3-methyl-2-penten-4-yn-1-ol, and a symmetrical dialdehyde has been discovered and applied to industrial production [8]. The synthetic AST is composed of a ratio of approximately 1:2:1 of (3*S*,3′*S*), (3*R*,3′*R*), and (3*R*,3′*S*) [8]. The structures of the AST isomers show distinct characteristics and, consequently, differences in bioactivity [9]. The antioxidant activity of the (3*S*,3′*S*) stereoisomer is higher than that of (3*R*,3′*R*), while the lowest antioxidant activity is found in the (3*R*,3′*S*) *meso*-form [10]. Due to low bioavailability, the synthetic product is currently not allowed for human consumption because of safety issues.

To reach and keep up with the increasing demand for the market of AST, efforts have been made to enhance the production of AST in some microorganisms through metabolic engineering. Previous studies have reported that the construction of the biosynthesis pathway for AST production was established in a strain of *Escherichia coli* that has been considered GRAS (Generally Recognized as Safe) and has been in commercial use in food industries. The strain resulted in the production of 5.8 mg/g DCW AST [11]. Recently, Lin et al. reported that a strain of genetically modified yeast (*Kluyveromyces marxianus*) obtained the ability of 3*S*,3′*S*-AST production without other optical isomers [12]. Two enzymes from algae, *β*-carotene ketolase (*bkt*) and hydroxylase (*hpchyb*), were included in the yeast for constructing a better AST biosynthesis pathway [12]. The yeast expressed both enzymes, and the resulting AST could respectively yield up to 3.125 and 5.701 mg/g DCW in a different medium, which turned it into the highest-produced microorganism in nature [12]. The investigation of the safety of the AST products from yeast was achieved by in vitro assay and animal models [13]. In DPPH scavenging analysis, the AST products showed a significant antioxidant ability. The two animal models, zebrafish and rat, showed no significant toxic effects. In addition, rats with lung cancer that were fed the AST products showed inhibition of metastasis in cancer cells and an increased survival rate [13]. Therefore, it is urgent to establish an optimal preparation method for yeast-produced AST.

However, other carotenoids with similar structure and polarity that are produced from the AST biosynthetic pathway, such as *β*-carotene, lutein, canthaxanthin, and zeaxanthin (Figure 1E–H), increase the difficulty of AST isolation [14]. Therefore, a simple method for removing the impurities in the isolation of high-purity 3*S*,3′*S*-AST should be developed.

Previous studies have mainly focused on two approaches, extraction and chromatography, which involve the development of an optimal extraction method and isolation of AST. The extraction methods of AST from *H. pluvialis* [15], *Phaffia rhodozyma* [16], and other microbial sources were extensively studied [17]. Industrial production is mainly based on maceration [18], supercritical extraction [19], ultrasound-assisted [20] or enzyme-assisted extraction [21] of carotenoids and AST [22]. These methods require large volumes of solvents, are time-consuming, and have low extraction efficiencies. However, enzyme-assisted extraction is not only a simpler and more efficient way, but it also uses less solvent because it can be reused.

Several methods of chromatography were applied to the isolation of AST from natural resources, including high-speed counter-current chromatography (HSCCC), simulated moving bed, and silica gel open columns. Du et al. used HSCCC to obtain AST from *P. rhodozyma* [23]. Liang et al. used a simulated moving bed to isolate AST [24]. However, there are several commonly encountered drawbacks in these isolation and purification processes, such as the need for large amounts of expensive gels as well as high volumes of solvents, time-consuming procedures, and limited scale-up potential. In addition, the procedures may not be suitable for the food and nutraceutical industries. Therefore, the development of a simple yet systematic and complete preparation for extraction, isolation, and purification of high-purity 3*S*,3′*S*-AST is necessary.

In this study, we developed a highly efficient method for extracting and isolating 3*S*,3′*S*-AST from the genetically modified yeast without gel column chromatography and gel filtration. The extraction yield of 3*S*,3′*S*-AST was enhanced with an enzymatic cell wall disruption. Carotenoid standards and various salts were used to monitor and modify the salt-assisted liquid-liquid extraction (SALLE), and AST was isolated successfully from carotenoid mixtures by forming complexes with metal ions. We further combined both enzyme- and salt-assisted methods and applied them to 3*S*,3′*S*-AST isolation from yeast, and highly purified 3*S*,3′*S*-AST products were obtained from this simple preparation. This developed method may replace column chromatography or gel filtration with a few steps of processes, simple equipment, and minimum volumes of solvents. Moreover, this method is more environmentally friendly as the enzymes, salts, and solvents can be recycled for reuse. These results will enable manufacturers to improve the 3*S*,3′*S*-AST production and provide benefits to human health.

## 2. Materials and Methods

**Figure 2:**
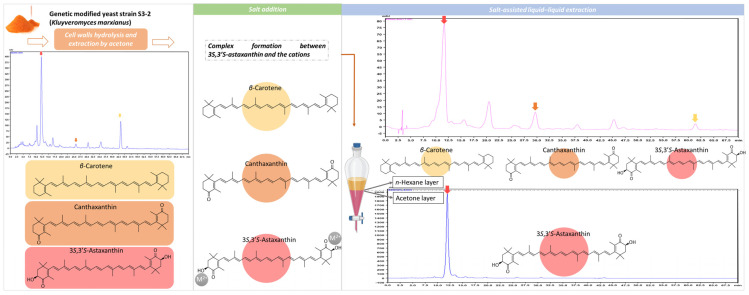
The scheme illustrating the isolation of high-purity 3*S*,3′*S*-astaxanthin from yeast.

The scheme for high-purity 3*S*,3′*S*-AST preparation is shown in Figure 2. First, the yeast powder was suspended in an enzyme solution. The mixture was incubated overnight to hydrolyze the cell walls to enhance the extraction efficiency and then filtered, and the mass was further extracted with acetone. The acetone extracts were dried using a rotary evaporator at 40 °C. The dried extracts were re-dissolved with acetone salt solution for cation chelation with 3*S*,3′*S*-AST. The acetone salt solution was then partitioned with *n*-hexane to isolate 3*S*,3′*S*-AST.

### 2.1. Chemicals

Four carotenoid standards (*β*-carotene ≥ 95%; canthaxanthin ≥ 95%; zeaxanthin ≥ 95%; and astaxanthin ≥ 97%) were obtained from Sigma-Aldrich (St. Louis, MO, USA). The salts were purchased from J.T. Baker and Sigma-Aldrich. Glucanex^®^ and hemicellulase were obtained from Sigma-Aldrich and FoodPro^®^ CBL from Chen-Ding Enterprises Co., Ltd. (Taipei, Taiwan). Acetone and *n*-hexane (LC grade) were from MERCK (Darmstadt, Germany). For chromatography analysis, LC-grade methanol (MeOH) was obtained from MERCK, and LC-grade methyl *tert*-butyl ether (MtBE) from Duksan Pure Chemicals (Ansan, South Korea). Both hydrochloric acid and ammonia solutions for pH value adjustment were bought from MERCK (Darmstadt, Germany). The tested salts for chelation were purchased from Alfa Aesar (Ward Hill, MA, USA), J.T. Baker, MERCK, and Sigma-Aldrich. The water was double distilled and deionized (≥18 MΩ·cm resistivity at 25 °C). All standard solutions were prepared using LC-grade acetone.

### 2.2. Materials

The genetically modified yeast *Kluyveromyces marxianus* was obtained in dried powder form from Trade Wind Biotech Co., Ltd. (Taipei, Taiwan). This strain was bioengineered with the technique called “promoter-based gene assembly and simultaneous overexpression” (PGASO) [25], which has been used to construct the carotenoid biosynthesis pathway in yeast for the purpose of scaling up the production of 3*S*,3′*S*-AST without other optical isomers [12]. The yeast and yeast-produced 3*S*,3′*S*-AST have passed the safety assessment to be nontoxic [26].

### 2.3. Enzyme-Assisted Extraction

Enzyme-assisted extraction was performed as in a previous report [22] with some modifications. In brief, the yeast extract was prepared as follows: 5 g of yeast powder was suspended in 100 mL enzyme solutions (FoodPro^®^ CBL, Glucanex^®^, and hemicellulose) in an appropriate concentration. The incubation time, temperature, and pH optimal for enzyme activity were selected according to those reported in the protocol provided by the suppliers. After incubation, the mixture was centrifuged at 4500 rpm for 10 min or filtered to collect the residues. The resulting residues were subsequently extracted with acetone. On completion of the extraction, the extracts were filtered to collect the supernatant and evaporated to yield a 3*S*,3′*S*-AST–rich extract.

### 2.4. Salt-Assisted Liquid-Liquid Extraction (SALLE)

Salt-assisted liquid-liquid extraction is based on the phase of water-immiscible organic solvents from water solutions by salt addition [27]. In this study, we simulated the SALLE system but adjusted the conditions for extraction solvents: the aqueous salt solution was replaced with acetone salt solution and the water-immiscible organic solvent, such as *n*-butanol, was replaced with *n*-hexane, which acted as an acetone-immiscible organic solvent. After salt addition, the acetone salt solution could be separated into two layers while partitioned with *n*-hexane. First, the salt was dissolved in acetone, and then the carotenoid samples were mixed with the acetone salt solution. Then, *n*-hexane was used as the partition solvent, which consisted of *n*-hexane/acetone salt solution (1:1, *v*/*v*), and the step was repeated 3 times. Finally, the acetone layer was collected for further analysis. After the SALLE procedure, the acetone layer was collected and evaporated. The dried extract was suspended in distilled water and kept at 4 °C to complete the desalting and precipitation. The precipitate was collected after filtering and washed with ethanol and *n*-hexane to remove water and impurities.

### 2.5. High-Performance Liquid Chromatography (HPLC)

The HPLC system (Shimadzu Inc., Kyoto, Japan) consisted of an SPD-M10A photodiode array detector (Shimadzu Inc., Kyoto, Japan), which was used for analysis. The following mobile phases were employed with the carotenoid reversed-phase column (4.6 mm in diameter × 250 mm in length, particle size of 5 μm; YMC, Tokyo, Japan): solvent A consisted of MeOH/MtBE/H_2_O (81:15:4, *v*/*v*/*v*), and solvent B consisted of MeOH/MtBE/H_2_O (7:90:3, *v*/*v*/*v*). The flow rate of the mobile phase was 1 mL/min with the following enhanced 70-min gradient elution of solvent B: 0% maintained for 5 min initially, reaching 50% in 60 min and returned to initial conditions within 1 min, for a 5-min re-equilibration. The column was at ambient temperature (ca. 25 °C), and detection was set at 480 nm. The carotenoids were prepared by being dissolved in acetone and confirmed by using chromatography, commercial reference products, and comparison of their spectra.

### 2.6. Nuclear Magnetic Resonance Spectroscopy (NMR)

One milligram of the AST standard was prepared for loading into the JEOL NM-ECS 400 NMR Spectrometer (JEOL Ltd., Tokyo, Japan) after being dissolved in 500 μL Acetone-*d*_6_ with salts to observe the changes of chelation. The parameter of the experiment was set at (1) the number of scans, 32; (2) relaxation time, 2 s; and (3) pulse program zg30. The results were examined by the MestReNova software. According to the residual proton resonances of the appropriate deuterated solvent (Acetone-*d*_6_), ^1^H NMR chemical shifts were reported.

### 2.7. Oxygen Radical Antioxidant Capacity (ORAC) Activity Assay

Three types of AST samples, raw material extracts, standards, and purified products, were prepared in 50 μg/mL using DMSO. The ORAC assay was performed with ORAC Assay Kit (ab233473) purchased from Abcam (Cambridge, UK) according to the method reported in the previous study [28]. A 1X assay diluent was diluted 1:4 with deionized water and mixed to homogeneity. While preparing the 1X fluorescein probe, the fluorescein probe was diluted 1:100 with 1X assay diluent and mixed to homogeneity. The 80 mg/mL free radical initiator solution had to be prepared fresh in 1X PBS. In a 96-well plate, 150 μL of the 1X fluorescein solution was added to the experimental wells. Additionally, the wells for blank were added with 25 μL Trolox solution. The AST samples were added with 25 μL to the sample wells. The amount of 25 μL of the free radical initiator solution was added into each well, and the reaction mixture was mixed by pipetting to ensure homogeneity. The sample and standard wells were read immediately with BioTek Cytation 5 cell imaging multimode reader (Agilent, Santa Clara, CA, USA) at 37 °C at Ex/Em = 480/520 nm.

The ORAC values are expressed as μM Trolox equivalents (μM TE), according to the following formula:

(1) $$\mathrm{ORAC}\;\left(\mu M\;\mathrm{TE}\right)=f\left[Trolox\right]\cdot \frac{\left(AUC_{sample}-AUC_{control}\right)}{\left(AUC_{Trolox}-AUC_{control}\right)}$$

where *f* is the dilution factor, and AUC is the area below the fluorescence decay curve of the sample, control, and Trolox, respectively. The AUC can be calculated from the following formula:

(2) $$\mathrm{AUC}=1+RFU_{1}/RFU_{0}+RFU_{2}/RFU_{0}+RFU_{3}/RFU_{0}+\cdots +RFU_{n}/RFU_{0}$$

where *RFU*_0_ is the relative fluorescence value of time point zero, and *RFU_n_* is the relative fluorescence value at time *n*.

### 2.8. Statistical Analysis

All data are presented as mean ± SD (standard deviation). The ORAC assay is independently analyzed in triplicate, and the results are analyzed using GraphPad Prism 6 (GraphPad Software, San Diego, CA, USA). The significance was established at *p* < 0.05.

## 3. Results and Discussion

### 3.1. Enzyme Selection for the Yeast Cell Wall Hydrolyzation

The genetically modified yeast contains multiple carotenoids, including *β*-carotene, canthaxanthin, and 3*S*,3′*S*-AST. It is significant to have a simple method to extract the carotenoids for the production of high-quality 3*S*,3′*S*-AST extracts. The yeast cells are composed of rigid cell walls that block the entry of organic solvents for extraction. The enzyme-assisted extraction method helps to hydrolyze the cell wall structure and expose intracellular components, thereby enhancing the yield of 3*S*,3′*S*-AST. A previous study has reported that glucanase and protease could attain the highest AST content after cell wall hydrolyzation [21]. Thus, it is important to find suitable enzymes to hydrolyze the cell walls, which can be used in the food and biomedical industries.

**Figure 3:**
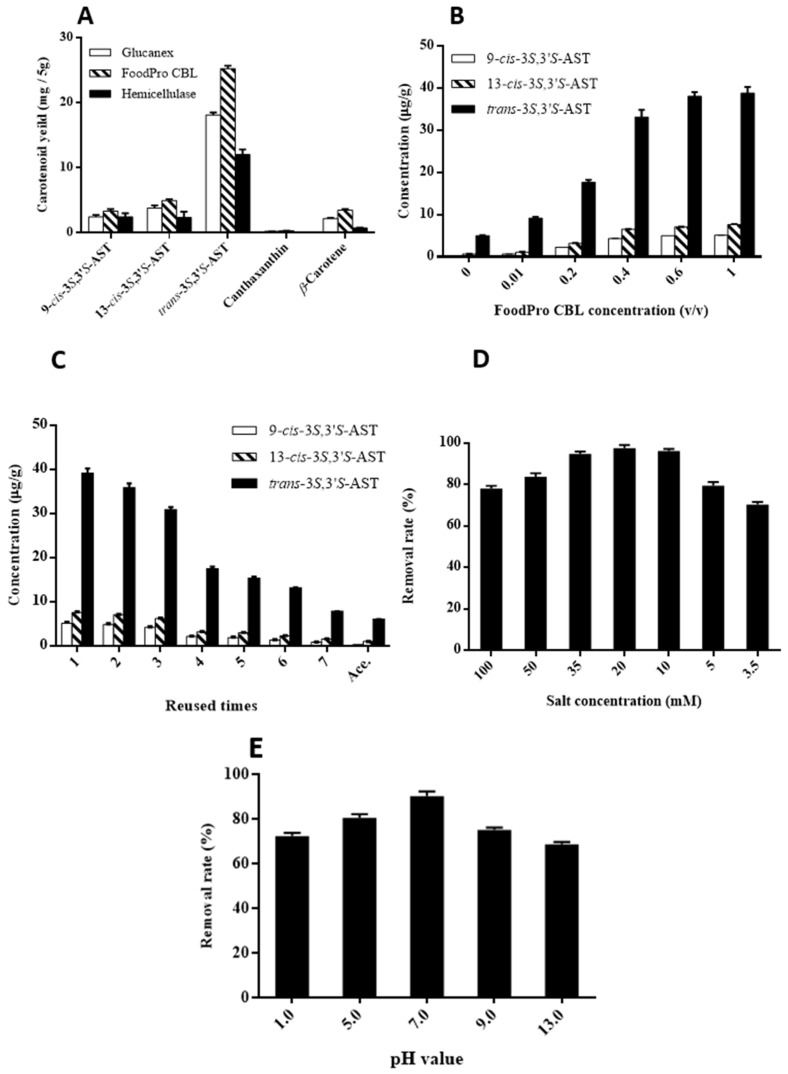
Extraction of carotenoids from *K. marxianus* by enzyme-assisted extraction with acetone. (**A**) Extraction of carotenoids after cell walls hydrolyzation with different enzymes. (**B**) Extraction of 3*S*,3′*S*-AST with different concentrations of FoodPro^®^ CBL. (**C**) Extraction of 3*S*,3′*S*-AST with different rounds of FoodPro^®^ CBL solution reusing. (**D**,**E**) Effects of impurities removal rate. (**D**) Salt concentration and (**E**) pH values.

In order to obtain a high yield of 3*S*,3′*S*-AST, three enzymes—FoodPro^®^ CBL (1%; *v*/*v*), Glucanex^®^ (1%; *w*/*w*), and hemicellulose (1%; *w*/*w*)—were used to hydrolyze the cell walls. As shown in Figure 3A, FoodPro^®^ CBL resulted in the highest extraction yield of 3*S*,3′*S*-AST, which was 25.28 ± 0.46 (mg/5 g). The best enzyme for 3*S*,3′*S*-AST and other carotenoids was determined to be FoodPro^®^ CBL using the enzyme-assisted extraction method.

The efficiencies of enhancing the yield of 3*S*,3′*S*-AST using different concentrations of FoodPro^®^ CBL were determined. As shown in Figure 3B, the yields of 9-*cis* and 13-*cis*-3*S*,3′*S*-AST were similar for 0.6% and 1% (*v*/*v*), while 1% provided the highest yield. The rounds for FoodPro^®^ CBL reusing were also determined. As shown in Figure 3C, although there was a significant drop in the fourth round due to a reduced cell wall hydrolyzation ability, but the yield of 3*S*,3′*S*-AST remained higher than maceration after reusing for seven rounds.

### 3.2. Salt Selection for the Chelation of 3S,3′S-astaxanthin

Previous studies have reported that cations could simply form complex formations with AST in organic solvents [29]. Therefore, twenty kinds of cations were tested for solubility in acetone. As shown in Table S1, the perchlorate salts were the only salt type that can dissolve in acetone, which indicated similar results to the previous reference [29]. Currently, the SALLE system can be formed by adding a moderate amount of perchlorate salts, and it is limited by the solubility of salt in acetone. Compared with other perchlorate salts, magnesium perchlorate [Mg(ClO_4_)_2_] may provide Mg^2+^ ions with a divalent cation and smaller size cation for chelating with the *α*-keto-hydroxyl group on the cyclohexane ring of AST. Thus, Mg(ClO_4_)_2_ was selected as the SALLE agent for subsequent studies.

**Figure 4:**
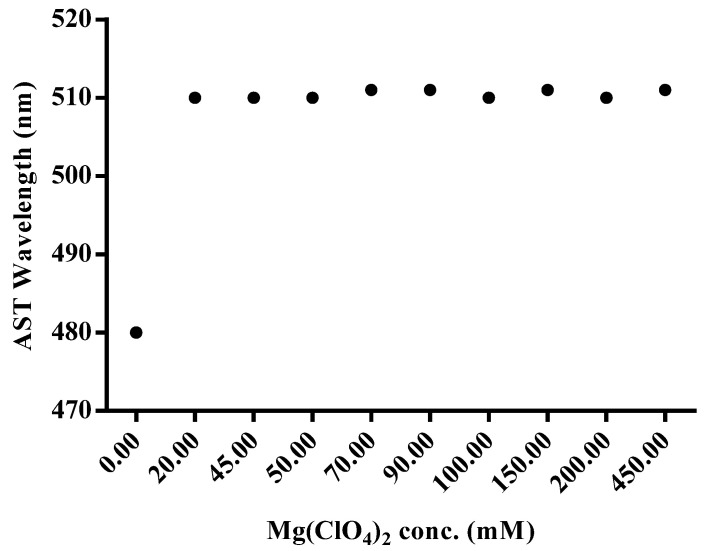
The wavelength shifting of the chelation of Mg(ClO_4_)_2_ and astaxanthin standard as a function of the concentration of Mg(ClO_4_)_2_.

The selection of an appropriate concentration of Mg(ClO_4_)_2_ is significant for the chelation between AST and Mg^2+^ ions. In this study, the effect of the concentration of Mg(ClO_4_)_2_ from 0 to 450 mM was investigated when 0.02 mM AST standard was used. As shown in Figure 4, when the concentrations of Mg(ClO_4_)_2_ were increased from 0 to 20 mM, the wavelength of AST showed an obvious redshift from 480 to 511 nm. In the ^1^H NMR, downfield chemical shifts of several protons were observed after the chelation between AST and Mg^2+^ ions at 20 mM of Mg(ClO_4_)_2_ in acetone solution (Figure S1). The chemical shift of 3-CH proton, which is close to a hydroxyl group, showed an obvious downfield shift (+0.76 ppm) from 4.29 ppm to 5.05 ppm, while both of the methyl groups, 1-CH_3_ and 2-CH_3_ protons, showed small changes of +0.08 ppm (Table 1). Thus, we selected 20 mM of Mg(ClO_4_)_2_ for the next SALLE procedure.

The reason for the increase in absorbance may be that the chelation between AST and Mg^2+^ ions provided a π-electron conjugation of cyclohexanone to polyene, thus giving a reasonable explanation for the redshift of absorbance [30]. The results of NMR measurement strongly indicate that the *α*-keto-hydroxyl group acts as the main functional site by utilizing the ion pairs, which act as electron donors in chelating with Mg^2+^ ions.

### 3.3. Isolation of Astaxanthin with SALLE from a Carotenoid Mixture

The pH value and salt concentration play an important role in the SALLE procedure, as they may affect the removal rate of impurities. The effect of seven different salt concentrations of Mg(ClO_4_)_2_ on the removal rate of impurities was studied. As shown in Figure 3D, the removal rate of impurities increased gradually with the increase of salt concentration from 3.5 to 20 mM, and thereafter decreased with the increase of the salt concentration from 35 to 100 mM. The best salt concentration was obtained at 20 mM, which can remove 97.5% of impurities. That is probably due to the conjugated double bonds of the hydrophobic carotenoids (*β*-carotene, canthaxanthin, and zeaxanthin) that start to interact with Mg^2+^ ions after the *α*-keto-hydroxyl group of astaxanthin is fully chelated, while the salt concentration sover 20 mM tends to stay in the hydrophilic phase.

The effect of five different pH values (pH 1.0, 5.0, 7.0, 9.0, and 13.0) of acetone solution on the removal rate of impurities was studied. It is known that carotenoids are sensitive to pH value, which can be isomerized or degraded by forming carotenoid radicals by exposure to acid or base [31]. As shown in Figure 3E, pH 7.0 exhibited the highest removal rate (90.3%) of impurities, and the removal rate decreased with the acidic and basic conditions. The reason may be that exposure to acids is thought to produce ion-pairs, and the carotenoids can be dissociated to form an unstable carotenoid carbocation [31]. The reason for the decrease in basic conditions may be that the carotenoids are degraded by alkaline hydrolysis [32]. Both acidic and basic conditions may increase other impurities, which may tend to stay in the hydrophilic phase. Based on the above experimental results, 20 mM salt concentration and pH 7.0 were selected for the SALLE procedure applied on yeast-produced 3*S*,3′*S*-AST.

### 3.4. Isolation of 3S,3′S-astaxanthin from Yeast Extract with SALLE

**Figure 5:**
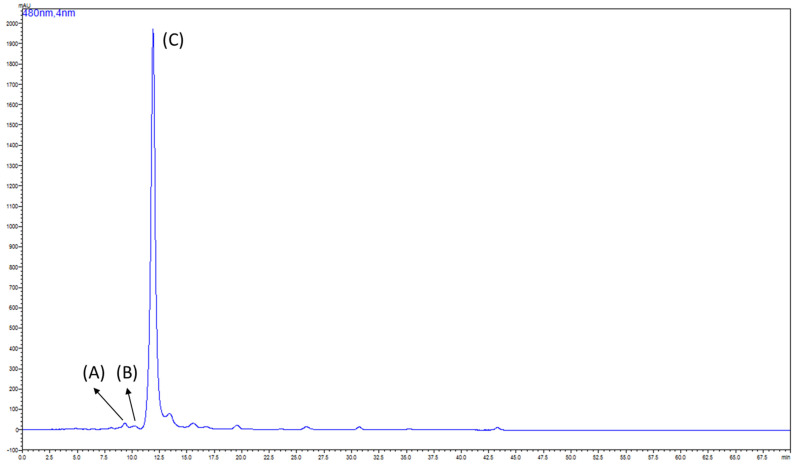
HPLC fingerprint of isolated product. The peaks were determined as (**A**) 9–*cis*–3*S*,3′*S*–astaxanthin; (**B**) 13–*cis*–3*S*,3′*S*–astaxanthin; and (**C**) *trans*–3*S*,3′*S*–astaxanthin. Detection was carried out at 480 nm.

In this study, the dry powder of yeast (*K. marxianus*) was used in the preparation method. After the cell walls were hydrolyzed by FoodPro^®^, the yeast protoplast was filtered and extracted with acetone. The acetone extract was evaporated and re-dissolved by 1 L 20 mM Mg(ClO_4_)_2_ acetone salt solution, and an even volume of *n*-hexane was used to initiate the following SALLE procedure. The acetone salt layer was collected and evaporated after the SALLE was repeated three times. Then, the extract was suspended in double-distilled water and kept at 4 °C for precipitation. The precipitate was filtered and washed with ethanol and *n*-hexane to remove remaining salt, water, and impurities. The HPLC fingerprint of the purified 3*S*,3′*S*-AST products are shown in Figure 5; the *trans*-3*S*,3′*S*-AST (peak C) appeared as the major component in comparison with two other minors (peaks A and B). In the UV/vis spectrum, peak A and peak B were determined as 9-*cis*-3*S*,3′*S*-AST and 13-*cis*-3*S*,3′*S*-AST, respectively [33]. The purity of 3*S*,3′*S*-AST was determined to be over 99% with HPLC analysis. Therefore, this new preparation method successfully purified 3*S*,3′*S*-AST from the yeast extract with SALLE and replaced gel filtration and column chromatography.

For the reasons mentioned in Section 3.2, the chelation between 3S,3′S-AST and Mg^2+^ ions caused 3*S*,3′*S*-AST tend to stay in the acetone salt solution. In contrast, other components without chelation may tend to stay in the hydrophobic layer. Similar to sodium chloride dissolved in water, magnesium perchlorate dissolved in acetone turns acetone into an ionic solution, which exhibits a stronger polar property. Therefore, when Mg(ClO_4_)_2_ is added to acetone, the simultaneous reaction of chelation between 3*S*,3′*S*-AST and Mg^2+^ ions as well as the enhancement of solubility of the complex formation leads to an efficient way in the isolation and purification of 3*S*,3′*S*-AST from the yeast extract.

Regarding the separation, isolation and purification of astaxanthin, column chromatography and gel filtration are the common methods reported in previous references. For example, Du et al. obtained astaxanthin from *P. Rhodozyma* by HSCCC and a two-phase solvent system composed of *n*-hexane/acetone/ethanol/water (1:1:1:1, *v*/*v*/*v*/*v*) followed by silica gel column chromatography, with the purity of astaxanthin reaching 99.0% [23]. Liang et al. designed a seven-zone simulated moving bed to obtain astaxanthin with nearly 90% recovery and purity [24]. Nonetheless, those methods involved a large volume of working solvent, stationary phase volume, and/or time-consuming processes. Therefore, this preparation method reported herein could have the potential for scaling up the production of high-purity 3*S*,3′*S*-AST in the near future.

### 3.5. Antioxidant Activity Determinations

Previous studies reported strong antioxidant activities of AST due to its unique structure that is able to transport electrons and neutralize free radicals [34]. In this study, we verified the antioxidative capability in inhibiting peroxy-radical-induced oxidation of low-purity raw extracts (ca. 0.25%) and final high-purity products (>99%) by an ORAC assay, which was determined by observing fluorescence changes (ORAC-FL) [28].

As a result, the high-purity 3*S*,3′*S*-AST products exhibited higher antioxidant capacity than the Trolox reference and AST standard through the ORAC-FL index values (Table 2), and the ORAC values were positively correlated with the purity of products. The high-purity 3*S*,3′*S*-AST products exhibited an antioxidant capacity that is more than 18.3 times as high as that of the low-purity raw extract, while the standard showed the value of only about 15.8 times as high. The results indicated that our preparation method could enhance the purity and antioxidant capacity of 3*S*,3′*S*-AST from low-purity raw extracts to final high-purity products.

The conjugated double bonds contained in AST act as strong antioxidants by reacting with free radicals to complete antioxidation [5]. However, other carotenoids, such as *β*-carotene and canthaxanthin, are also known as potent antioxidants with conjugated double bonds. The structural differences of AST are such that it contains hydroxyl and keto groups on the terminal cyclohexane ring at the para position, which may help AST to block the oxidative reaction. Our preparation method successfully retained the strong antioxidant activity products and 3*S*,3′*S*-AST and removed low antioxidant-activity impurities (*β*-carotene and canthaxanthin, etc.) powerfully.

## 4. Conclusions

In summary, this is the first time that a new preparation method was developed to yield high-purity 3*S*,3′*S*-AST from genetically modified yeast (*K. marxianus*), with a combination of enzyme-assisted extraction and SALLE by cation chelation. The method successfully achieved overall improved extraction yield (25.28 ± 0.46 mg/5 g) and purity (>99%) of the 3*S*,3′*S*-AST products. The antioxidant capacity of high-purity 3*S*,3′*S*-AST products showed higher (>18.3 times) values than that of low-purity raw material extracts. A combination of enzyme-assisted extraction and SALLE for high-purity 3*S*,3′*S*-AST production can be used to replace both column chromatography and gel filtration that is currently used in industry. The new combination preparation method provides a great potential to be scaled up in manufacturing high-quality 3*S*,3′*S*-AST and other individual bioactive ingredients in industries in the future.

Despite the new preparation method of 3S,3′S-AST being able to reach high purity, the optimization for each procedure is still undergoing. In the cell wall hydrolysis procedure, the hydrolysis ability of FoodPro^®^ CBL was decreased after being reused for seven rounds. The reason may be because the carbohydrates, which were hydrolyzed from the yeast cell wall that dissolved in an aqueous solution, reached a saturated condition. To keep the hydrolysis activity, we are currently working on replacing the aqueous solution with distilling the concentration, which may help enhance the solubility of carbohydrates. As for the SALLE procedure, despite the product’s high purity, it may contain several perchlorates, which were added in excess for chelation because a considerable amount of the salt remained in the products. To avoid toxic solvents and salts remaining in the extract, the edible substances are being tested to replace the toxic ones. We look forward to our optimized preparation method that can be applied in food and pharmaceutical industries with low-cost, large-scale, continuous preparation and the quantitative analysis of high-quality, high-efficiency 3S,3′S-AST.

## Figures and Tables

**Table 1:** The changes in the ^1^H NMR (400 MHz) chemical shift (in ppm) of astaxanthin protons in acetone and in the presence of 20 mM Mg(ClO_4_)_2_.

	3–CH	3–OH	=CH–	1–CH_3_	2–CH_2_
Standard	4.29	3.97	6.35–6.80	1.21, 1.35	2.21
Complexes	5.05 (+0.76)	Merged in H_2_O	6.42–6.85	1.29 (+0.08)	2.29 (+0.08)

**Table 2:** Antioxidant capacity by the ORAC assay in various astaxanthin. Three types of astaxanthin: raw material extract, standard from Sigma-Aldrich Co., and purified products.

Samples	ORAC-FL Index ^(1)^	ORAC (μM TE)	ORAC Fold Increase
Raw material extracts	1.269 ± 0.024	136.50 ± 18.48	-
Purified products	3.370 ± 0.037	2501.66 ± 123.91	18.3
Purchased standard	3.066 ± 0.119	2161.33 ± 178.69	15.8

FL, relative fluorescence intensity. ^(1)^ Values are the mean ± SD deviation of three replicate experiments of each sample.

## Data Availability

Data are contained within the article.

## Supplementary Materials

The following supporting information can be downloaded at: https://www.mdpi.com/article/10.3390/antiox12040875/s1, Figure S1: The chelation between astaxanthin and the Mg(ClO_4_)_2_ in acetone solution. The 1H NMR spectra of astaxanthin in the absence and the presence of 40 mM Mg(ClO_4_)_2_; Table S1: List of cations tested for complex formation with 1 mM astaxanthin standard solution.

## Abbreviations

AST, astaxanthin; SALLE, salt-assisted liquid-liquid extraction; HPLC, high-performance liquid chromatography; NMR, nuclear magnetic resonance; ORAC, oxygen radical antioxidant capacity.

## References

[1] Yang C., Zhang H., Liu R., Zhu H., Zhang L., Tsao R. (2017). Bioaccessibility, cellular uptake, and transport of astaxanthin isomers and their antioxidative effects in human intestinal epithelial Caco-2 cells. J. Agric. Food Chem..

[2] Gammone M.A., Riccioni G., D’Orazio N. (2015). Marine carotenoids against oxidative stress: Effects on human health. Mar. Drugs.

[3] Valenti M.T., Perduca M., Romanelli M.G., Mottes M., Carbonare L.D. (2020). A potential role for astaxanthin in the treatment of bone diseases. Mol. Med. Rep..

[4] Du X., Wang X., Bai M., Liu S., Huang G., Zhang Q., Ni H., Chen F. (2020). A Quantitative Analysis Model Established to Determine the Concentration of Each Source in Mixed Astaxanthin from Different Sources. Molecules.

[5] Guerin M., Huntley M.E., Olaizola M. (2003). *Haematococcus* astaxanthin: Applications for human health and nutrition. Trends Biotechnol..

[6] Wayama M., Ota S., Matsuura H., Nango N., Hirata A., Kawano S. (2013). Three-dimensional ultrastructural study of oil and astaxanthin accumulation during encystment in the green alga *Haematococcus pluvialis*. PLoS ONE.

[7] Johnson E.A., An G.-H. (1991). Astaxanthin from microbial sources. Crit. Rev. Biotechnol..

[8] Ashford R.D. (2011). Ashford’s Dictionary of Industrial Chemicals.

[9] Sun W., Xing L., Lin H., Leng K., Zhai Y., Liu X. (2016). Assessment and comparison of in vitro immunoregulatory activity of three astaxanthin stereoisomers. J. Ocean Univ. China.

[10] Liu X., Luo Q., Cao Y., Goulette T., Liu X., Xiao H. (2016). Mechanism of different stereoisomeric astaxanthin in resistance to oxidative stress in *Caenorhabditis elegans*. J. Food Sci..

[11] Wang C.W., Oh M.K., Liao J.C. (1999). Engineered isoprenoid pathway enhances astaxanthin production in *Escherichia coli*. Biotechnol. Bioeng..

[12] Lin Y.J., Chang J.J., Lin H.Y., Thia C., Kao Y.Y., Huang C.C., Li W.H. (2017). Metabolic engineering a yeast to produce astaxanthin. Bioresour. Technol..

[13] Tseng C.-C., Lin Y.-J., Liu W., Lin H.-Y., Chou H.-Y., Thia C., Wu J.H., Chang J.-S., Wen Z.-H., Chang J.-J. (2020). Metabolic engineering probiotic yeast produces 3S, 3′ S-astaxanthin to inhibit B16F10 metastasis. Food Chem. Toxicol..

[14] Schoefs B., Rmiki N.E., Rachadi J., Lemoine Y. (2001). Astaxanthin accumulation in *Haematococcus* requires a cytochrome P450 hydroxylase and an active synthesis of fatty acids. FEBS Lett..

[15] Ranga R., Sarada A.R., Baskaran V., Ravishankar G.A. (2009). Identification of carotenoids from green alga *Haematococcus pluvialis* by HPLC and LC-MS (APCI) and their antioxidant properties. J. Microbiol. Biotechnol..

[16] Mussagy C.U., Pereira J.F.B., Dufossé L., Raghavan V., Santos-Ebinuma V.C., Pessoa A. (2021). Advances and trends in biotechnological production of natural astaxanthin by *Phaffia rhodozyma* yeast. Crit. Rev. Food Sci. Nutr..

[17] Rao A.R., Reddy R.L.R., Baskaran V., Sarada R., Ravishankar G.A. (2010). Characterization of Microalgal Carotenoids by Mass Spectrometry and Their Bioavailability and Antioxidant Properties Elucidated in Rat Model. J. Agric. Food Chem..

[18] Michelon M., de Matos de Borba T., da Silva Rafael R., Burkert C.A.V., de Medeiros Burkert J.F. (2012). Extraction of carotenoids from *Phaffia rhodozyma*: A comparison between different techniques of cell disruption. Food Sci. Biotechnol..

[19] Zaghdoudi K., Framboisier X., Frochot C., Vanderesse R., Barth D., Kalthoum-Cherif J., Blanchard F., Guiavarc’h Y. (2016). Response surface methodology applied to Supercritical Fluid Extraction (SFE) of carotenoids from Persimmon (*Diospyros kaki* L.). Food Chem..

[20] Singh D., Barrow C.J., Mathur A.S., Tuli D.K., Puri M. (2015). Optimization of zeaxanthin and β-carotene extraction from *Chlorella saccharophila* isolated from New Zealand marine waters. Biocatal. Agric. Biotechnol..

[21] Machado F.R., Trevisol T.C., Boschetto D.L., Burkert J.F., Ferreira S.R., Oliveira J.V., Burkert C.A.V. (2016). Technological process for cell disruption, extraction and encapsulation of astaxanthin from *Haematococcus pluvialis*. J. Biotechnol..

[22] Saini R.K., Keum Y.-S. (2018). Carotenoid extraction methods: A review of recent developments. Food Chem..

[23] Du X., Dong C., Wang K., Jiang Z., Chen Y., Yang Y., Chen F., Ni H. (2016). Separation and purification of astaxanthin from *Phaffia rhodozyma* by preparative high-speed counter-current chromatography. J. Chromatogr. B.

[24] Liang R.C., Bao X.Q., Sung L., Lin C.H., Liang M.T. (2017). The design and operation of a simulated moving bed for the separation of intermediate retention components from a multi-component feedstock with a very strong retention component. Adsorption.

[25] Chang J.J., Ho C.Y., Ho F.J., Tsai T.Y., Ke H.M., Wang C.H., Chen H.L., Shih M.C., Huang C.C., Li W.H. (2012). PGASO: A synthetic biology tool for engineering a cellulolytic yeast. Biotechnol. Biofuels.

[26] Samuel S.Y., Wang H.M.D., Huang M.Y., Cheng Y.S., Chen J.R., Li W.H., Chang J.J. (2022). Safety Assessment of 3S, 3′S Astaxanthin Derived from Metabolically Engineered *K. marxianus*. Antioxidants.

[27] Ramos R.M., Valente I.M., Rodrigues J.A. (2014). Analysis of biogenic amines in wines by salting-out assisted liquid–liquid extraction and high-performance liquid chromatography with fluorimetric detection. Talanta.

[28] Ou B., Hampsch-Woodill M., Prior R.L. (2001). Development and Validation of an Improved Oxygen Radical Absorbance Capacity Assay Using Fluorescein as the Fluorescent Probe. J. Agric. Food Chem..

[29] Polyakov N.E., Focsan A.L., Bowman M.K., Kispert L.D. (2010). Free radical formation in novel carotenoid metal ion complexes of astaxanthin. J. Phys. Chem. B.

[30] Chen C.S., Wu S.H., Wu Y.Y., Fang J.M., Wu T.H. (2007). Properties of astaxanthin/Ca^2+^ complex formation in the deceleration of cis/trans isomerization. Org. Lett..

[31] Boon C.S., McClements D.J., Weiss J., Decker E.A. (2010). Factors influencing the chemical stability of carotenoids in foods. Crit. Rev. Food Nci. Nutr..

[32] Liu Y., Xu M.J., Canfield L.M. (1998). Enzymatic hydrolysis, extraction, and quantitation of retinol and major carotenoids in mature human milk 11Supported by grants from Wyeth Ayerst Nutritionals and International Life Science Institute (ILSI). J. Nutr. Biochem..

[33] Zhao L., Chen F., Zhao G., Wang Z., Liao X., Hu X. (2005). Isomerization of trans-astaxanthin induced by copper (II) ion in ethanol. J. Agric. Food Chem..

[34] Grosso C., Valentão P., Ferreres F., Andrade P.B. (2015). Alternative and Efficient Extraction Methods for Marine-Derived Compounds. Mar. Drugs.

