# Supplementary material for: High-Purity Bioactive Ingredient—3S,3′S-Astaxanthin: A New Preparation from Genetically Modified Kluyveromyces marxianus without Column Chromatography and Gel Filtration
Source: Antioxidants (Basel). 2023 Apr 4;12(4):875. doi: 10.3390/antiox12040875 (PMC10135142; doi:10.3390/antiox12040875)
Supplement: Supplementary file 1 [file antioxidants-12-00875-s001.zip › antioxidants-2316991-supplementary.pdf]

Table S1. List of cations tested for complex formation with 1 mM astaxanthin standard solution.

Salt	Ion	Formula	Solubility in Acetone
$Al(ClO_4)_3$	Aluminum	Al^{3+}	Soluble
NH_4NO_3	Ammonium	NH_4^+	Insoluble
$Ca(ClO_4)_2$	Calcium	Ca^{2+}	Soluble
$CoBr_2$	Cobalt(II)	Co^{2+}	Insoluble
$CuCl_2$	Copper(II)	Cu^{2+}	Insoluble
$Fe(ClO_4)_2$	Iron(II)	Fe^{2+}	Soluble
$MnSO_4$	Manganese(II)	Mn^{2+}	Insoluble
$Mn(acac)_3$	Manganese(III)	Mn^{3+}	Insoluble
$Mg(ClO_4)_2$	Magnesium(II)	Mg^{2+}	Soluble
$NiBr_2$	Nickel(II)	Ni^{2+}	Insoluble
$VOSO_4$	Oxovanadium(IV)	VO^{2+}	Insoluble
KCl	Potassium	K^+	Insoluble
KH_2PO_4			Insoluble
$AgNO_3$	Silver	Ag^+	Insoluble
Na_2CO_3	Sodium	Na^+	Insoluble
Na_2HPO_4			Insoluble
NaH_2PO_4			Insoluble
Sodium citrate			Insoluble
$SnSO_4$	Tin(II)	Sn^{2+}	Insoluble
$Sn(CH_3COO)_4$	Tin(IV)	Sn^{4+}	Insoluble
VCl_3	Vanadium(III)	V^{3+}	Insoluble
VCl_5	Vanadium(V)	V^{5+}	Insoluble
$Y(NO_3)_3$	Yttrium(III)	Y^{3+}	Insoluble
$Zn(ClO_4)_2$	Zink	Zn^{2+}	Soluble

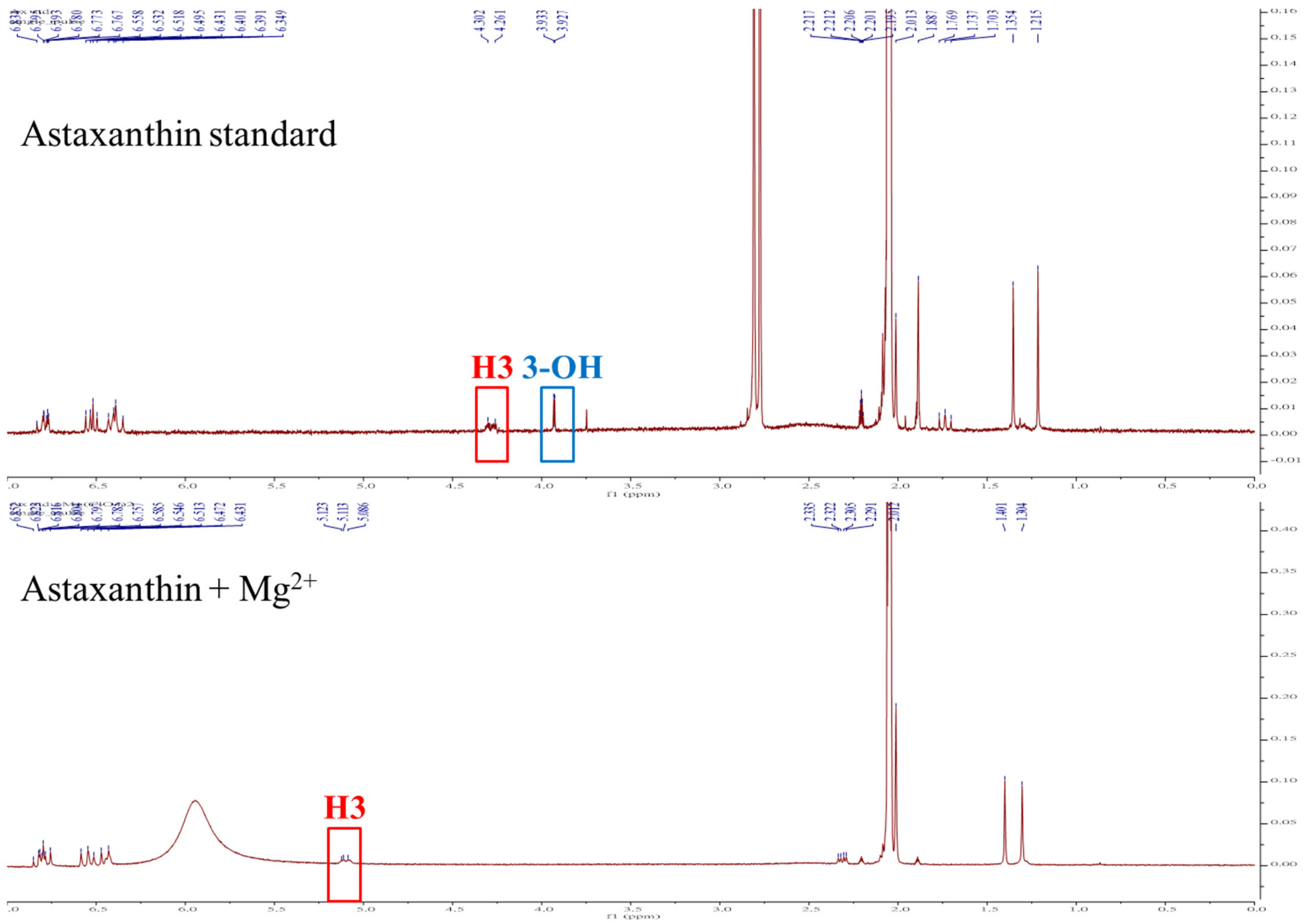

Figure S1. The chelation between astaxanthin and the $Mg(ClO_4)_2$ in acetone solution. The ^1H NMR spectra of astaxanthin in the absence and the presence of 40 mM $Mg(ClO_4)_2$.
